# Inverted Meckel's Diverticulum Simulating Pedunculated Polyp as a Lead Point for Ileoileal Intussusception in a Child

**Published:** 2013-01-01

**Authors:** Bilal Mirza

**Affiliations:** Department of Pediatric Surgery, The Children's Hospital and the Institute of Child Health Lahore, Pakistan

**Dear Sir,**

 Most of the intussusceptions in infants are idiopathic in nature. The incidence of intussusception decreases as the age advances. On the contrary, the frequency of lead point (secondary cause) increases with age. Meckel's diverticulum is the commonest anomaly of small bowel. There is 5% incidence of complications in patients having Meckel's diverticulum. Inverted Meckel's diverticulum, as a lead point for intussusception, is a rare entity and commonly reported in adults. Pediatric cases are scarcely reported in literature [1-4]. 

A 2-year-old girl presented with one day history of abdominal distension, bilious vomiting and not passing stool. There was no preceding history of significant illness. The patient was vitally stable. Abdomen was distended with tenderness on right side. Digital rectal examination revealed an empty rectum. Abdominal radiograph showed air fluid levels. Ultrasound of abdomen gave an impression of intussusception. Laboratory investigations were within normal limits. Patient was managed with intravenous fluids, prophylactic antibiotics, nasogastric decompression, and analgesics. Exploratory laparotomy was performed within 3 hours of presentation that revealed an ileoileal intussusception which was manually reduced. A polyp like pedunculated growth was palpable at about 30 cm proximal to ileocecal valve. An enterotomy was performed for polypectomy; however, the pedunculated growth had a pale tissue at the tip giving a suspicion of heterotopic tissue (Fig. 1). The involved segment of the ileum was resected and end to end hand sewn anastomosis was performed. The polypoid growth was inspected carefully and with a suspicion of inverted Meckel's diverticulum, reduced back confirming the diagnosis (Fig. 2). The patient had an uneventful recovery. She remained well at one year follow-up (1 year). Histopathology of the specimen revealed ectopic pancreatic tissue in the inverted Meckel's diverticulum.

**Figure:**
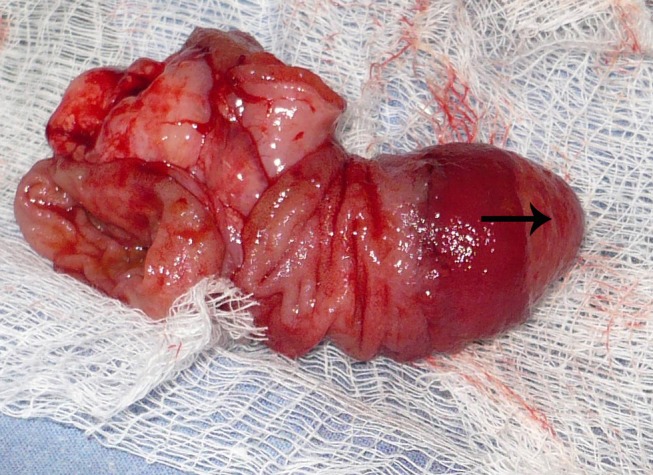
Figure 1: The specimen after resection showing a pedunculated polyp like growth with an ectopic tissue at the tip (arrow).

**Figure:**
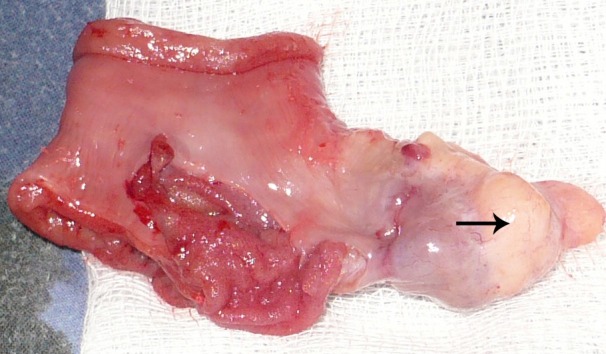
Figure 2: The polypoid growth after being reduced. Ectopic tissue at the tip is well appreciable (arrow).

Pantongrag-Brown et al [2] found that 21% of Meckel's diverticulae were inverted, of which 72% presented with small bowel intussusception; however, overall incidence of intussusceptions due to Meckel's diverticulum is about 4%. The exact mechanism of inversion of Meckel's diverticulum is not known but it is believed to result from hyperperistalsis generated due to irritation of enteric nervous system by activity of the ectopic tissues which is found in more than 50% cases of Meckel's diverticulum. In more than 85% of cases with heterotopy, gastric mucosa is present; pancreatic tissue is rarely reported in inverted Meckel's diverticulum as a lead point for intussusception. Meckel's scan, radio labeled RBC scan, superior mesenteric angiography, and CT scan are important diagnostic tools. On CT scan, a mass located in the distal small bowel, having a collar of soft tissue attenuation around a central area of fat attenuation, gives strong suspicion of inverted Meckel's diverticulum [1-4].

I believe that inversion of Meckel's Diverticulum could be a normal phenomenon in persons having it. It could revert, possibly, in the majority without being symptomatic. In few cases, inversion could advance to the extent which results in intussusception. Rarely, it could fix inside the bowel due to mucosal inflammation and swelling which may preclude the reversion; such inversion presents as bleeding per rectum (ectopic tissue), pain abdomen, intestinal obstruction secondary to intussusception (commonly) or mass (in case of hypertrophy) [1-5]. This theory plausibly explains the various presentations of inverted Meckel's diverticulum however further studies are required for scientific evaluation of this hypothesis. In cases where inversion advances to intussusception, Meckel's diverticulum could be reduced back along with the rest of invaginated bowel. In the index case, inverted Meckel's diverticulum could not be reduced with the invaginated bowel indicating a fixed inversion of Meckel's diverticulum. It simulated a pedunculated polyp, however after excision, the suspicion of ectopic tissue and careful dissection of the specimen made us identify it.

## Footnotes

**Source of Support:** Nil

**Conflict of Interest:** None declared
